# Low-Grade Oncocytic Tumor: Report of Two Cases of An Emerging Entity in the Spectrum of Oncocytic Renal Neoplasms

**DOI:** 10.5146/tjpath.2021.01549

**Published:** 2022-05-19

**Authors:** Divakar Sharma, Trupti Pai, Gagan Prakash, Sangeeta Desai, Santosh Menon

**Affiliations:** Departments of Pathology, Tata Memorial Centre, Homi Bhabha National Institute, Mumbai, India; Departments of Uro-Oncology, Tata Memorial Centre, Homi Bhabha National Institute, Mumbai, India

**Keywords:** Low-grade oncocytic tumor, Immunohistochemistry, Oncocytic renal tumors, Emerging renal tumors

## Abstract

Low-grade Oncocytic Tumor (LOT) of kidney is an emerging neoplasm that forms an important differential diagnosis in a spectrum of entities with oncocytic morphology. It has overlapping features with renal oncocytoma and eosinophilic variant of chromophobe renal cell carcinoma, but with distinct clinical, histomorphological and immunohistochemical features. LOT exhibits characteristic low grade oncocytic morphology with a CD117 negative/CK7 positive immunophenotype. Herein, we describe two cases of this emerging entity, LOT, with emphasis on the diagnostic aspects, including the histomorphology, immunoprofile and discussion on the close differentials.

## INTRODUCTION

Renal neoplasms with oncocytic/eosinophilic morphology are a growing list of entities and their diagnosis is sometimes challenging. Renal oncocytoma (RO) and eosinophilic variant of chromophobe renal cell carcinoma (eChRCC) are the most common tumors in this category. However, many cases do not fit into the gamut of existing classified entities. Low-grade Oncocytic Tumor (LOT) of kidney is a provisionally named emerging renal neoplasm that is not as yet included in the WHO classification of Renal tumors (1). These are low grade tumors with oncocytic features, albeit with absence of nuclear features of eChRCC and a characteristic CK7 positive/CD117 negative immunoprofile. Ultrastructural features of numerous mitochondria are seen in the cytoplasm of LOT (hence the designation ‘oncocytic’), similar to RO, as against numerous microvesicles with mitochondria that are seen in eChRCC (1,2). Additionally, karyotypic profile of LOT is also in variance with either RO or eChRCC.

LOT is an addition to the other recently described acronymic entities that include eosinophilic solid and cystic renal cell carcinoma (ESC RCC) and high-grade oncocytic renal tumor (HOT), all having distinct morphological and immunophenotypic profile (3). Recognition of these entities and their distinction from already characterized renal tumors is important. Herein, we present the clinicopathological features of two cases of this recently described entity of LOT.

## CASE REPORT

### Case 1

A 65-year male without any comorbidities presented with lower urinary tract symptoms like increased urinary frequency without any abdominal or flank pain. Ultrasonography revealed a left renal hyperechoic mass measuring 3.1x2.5 cm. Abdominal contrast enhanced computerized tomography (CECT) revealed a solitary, 2.7x1.7x1.4 cm exophytic lesion in the upper and mid-pole of the left kidney. Metastatic workup was negative. Left partial nephrectomy was performed.

**Figure 1:**
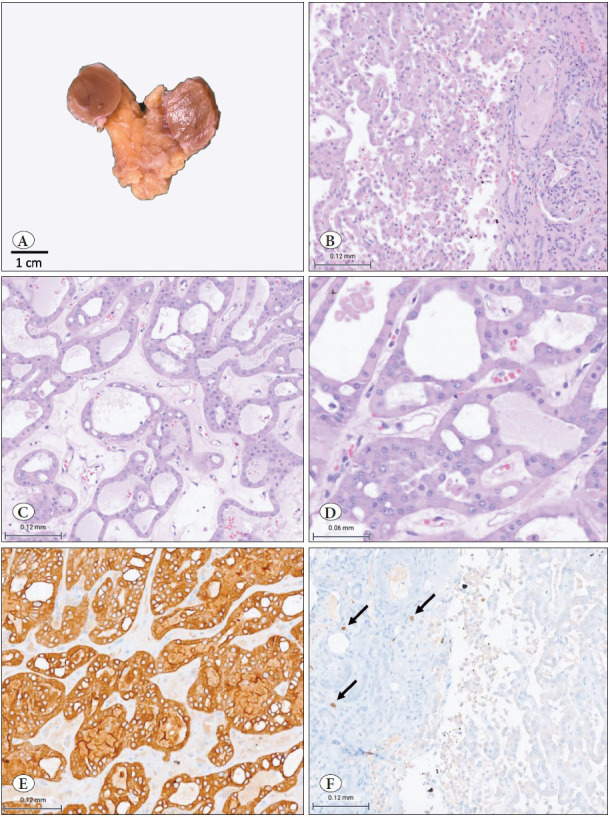
**Case 1: A)** Grossly, cut surface revealed a well-circumscribed, tan-brown, gelatinous and firm tumor. **B,C,D)** Microscopically, the tumor had an irregular interface with the adjacent renal parenchyma with no capsule (B; H&E; 200x). Tubuloreticular growth (C; H&E; 200x) with low grade oncocytic features (D; H&E; 400x) were observed. **E,F)** Immunohistochemically, diffuse CK7 positivity was seen (E; IHC; 200x), while CD117 was negative (F; IHC; 200x). Mast cells as internal control in the adjacent renal parenchyma were immunoreactive (F; arrows).

Gross findings revealed a well-circumscribed tumor measuring 2 cm in the greatest dimension (pT1a). The tumor had a tan brown, gelatinous and firm cut surface. No central scar, hemorrhage or necrosis was seen (Figure 1A).

On microscopy, the tumor was unencapsulated and had an irregular interface with compressed renal parenchyma at the periphery. The tumor had a solid, tubulocystic and focal cribriform pattern. Tumor cells were monomorphic, cuboidal in shape with moderate amount of oncocytic cytoplasm and round centrally placed nuclei and focal distinct nucleoli (WHO/ISUP grade equivalent was ISUP grade 2). There were no prominent cell membranes, no nuclear membrane irregularities or raisinoid nuclei. (Figure 1B-D). There was no appreciable mitotic activity or coagulative necrosis. Perinephric fat and Gerota’s fascia were free of tumor. TNM stage was pT1aNxMx.

On immunohistochemistry (IHC), the tumor cells were strongly and diffusely positive for CK7, while being negative for CD117 (Figure 1E,F), vimentin and Alpha-methylacyl-CoA racemase (AMACR). E-cadherin staining showed a diffuse membranous pattern and EMA showed focal apical staining.

Based on these features, a diagnosis of low-grade oncocytic tumor of kidney was given. Patient did not receive any adjuvant treatment. With a follow-up of 12 months till date, the patient is disease free and asymptomatic.

### Case 2

Case 2 was a referral case and no clinical details were available. We received an outside operated right radical nephrectomy specimen of a 59-year male patient for histopathological analysis.

**Figure 2:**
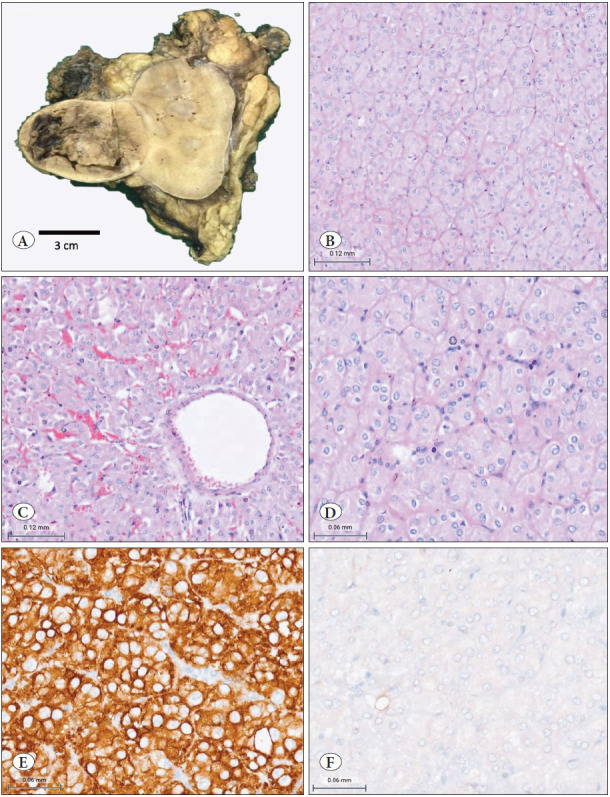
**Case 2: A)** Grossly, tumor cut surface was solid, grey-white to tan, soft in consistency with focal areas of hemorrhage **B,C,D)** Microscopically, a solid growth pattern was observed (B; H&E; 200x) with areas of perivascular growth and foci of hemorrhage (C; H&E; 200x). On high power, many oncocytic cells showed perinuclear halos with focal infiltrate of lymphocytes (D; H&E; 400x). **E,F)** Immunohistochemically, diffuse CK7 positivity was seen in the tumor cells (E; IHC; 400x) while these were negative for CD117 (F; IHC; 400x).

On gross examination, the tumor was well circumscribed, at midpole, predominantly cortical based, with medullary involvement. Tumor dimensions were 5.5x5x4 cm with a grey white to tan brown, soft cut surface and focal central areas of hemorrhage. No central scar or necrosis was observed (Figure 2A). The tumor was confined to the kidney; and the pelvicalyceal system, renal sinus, perinephric fat, Gerota’s fascia and all cut margins (renal artery, renal vein and ureter) were free.

On microscopy, the tumor was sharply demarcated with the adjacent renal parenchyma and had a predominantly solid and nested growth pattern. The tumor cells had uniform oncocytic cytoplasm with homogenous round to oval nuclei (WHO/ISUP grade equivalent was ISUP grade 1). Focal perinuclear halo were noted in many areas; however, similar to case 1 there were no nuclear membrane irregularities or raisinoid nuclei (Figure 2B-D). There was no mitotic activity or coagulative tumor necrosis. Four hilar nodes identified were all negative for metastases. TNM stage was pT1bN0Mx.

On IHC, the tumor cells were strongly and diffusely positive for CK7, while being negative for CD117 (Figure 2E,F), CD10, AMACR, Synaptophysin and Chromogranin. A diagnosis of low-grade oncocytic tumor of kidney was given. No follow-up details were available for this case.

## DISCUSSION

Oncocytic tumors with atypical or borderline morphology and immunoprofile that do not fit the criteria of existing defined entities are often reported descriptively as ‘Oncocytic renal neoplasm’ and comment is made regarding their risk or malignant potential (4). Trpkov et al. recently published a series of 28 cases of renal oncocytic tumor from 4 major institutions with characteristic low grade morphology and typical CK7 positive/CD117 negative immunoprofile, and designated such tumors as LOT after detailed histomorphological and array comparative genomic hybridization (aCGH) findings (1).

In this series by Trpkov et al. the median age of the patients was 66 years with a female preponderance. In our study, both were male patients, one 65 years old (Case 1) and the other 59 years old (Case 2). In concordance with the literature, there was no syndromic association.

On gross examination, both tumors had circumscribed borders; however, they were unencapsulated and had a brownish, solid cut surface, without necrosis as is seen in the previous studies. The majority of the cases (88%) belonged to stage 1 with a median tumor size of 3 cm (1). Similarly, both of our cases also were pT1, case 1 with 2 cm tumor size, while in Case 2, tumor size was 5.5 cm. Both were restricted to the kidney, without involvement of the perinephric fat, renal sinus or Gerota’s fascia. Regional nodes were negative for metastases (Case 2).

Microscopically, both the cases had a solid, compact tubulocystic pattern. As reported previously (1), the characteristic finding of loose reticular arrangement of tumor cells within the edematous stroma was observed in Case 1. This differentiates it histologically from oncocytoma, which shows compact tumor island and nests of tumor cells within the hypocellular stroma. Lymphocytic aggregates described in the literature were not observed in either of our cases (1). However, focal intercellular sprinkling of lymphocytes was observed amidst tumor cells in Case 2 (Figure 2D). Tumor cells had low grade oncocytic morphology without any nuclear membrane irregularities, multinucleation, or nuclear pleomorphism, which differentiates the cases from eChRCC.

On IHC, both tumors had consistent findings of strong diffuse CK7 positivity and CD117 negativity. The salient features of LOT with its two main close differentials (RO and eChRCC) are discussed in Table 1. IHC workup with CK7 and CD117 is commonly used to distinguish these oncocytic entities. While RO and eChRCC are diffusely positive for CD117, and negative or very focally positive for CK7, LOT shows consistent CK7 positive/CD117 negative pattern as seen in both of our cases, while Hybrid oncocytoma-chromophobe tumors (HOCT) are invariably CD117 positive (5). Petersson et al. have comprehensively described ‘sporadic’ HOCT in their case series of 14 cases (6). However, most of these tumors were positive for CK7 and negative or focally marked with CD117. In all probability, we believe these tumors were also cases of LOT that have been designated as HOCT.

**Table 1:** Salient features of LOT, Renal Oncocytoma and eosinophilic variant of ChRCC (1,2,7).

** **	**LOT**	**Oncocytoma**	**eChRCC**
**Microscopy**			
a) Pattern	Solid, compact nests with focal tubuloreticular growth. Frequently edematous stroma with loose cell growth	Solid, Solid-nested or rarely tubulo-cystic. Frequently loose hypocellular stroma	Solid, solid-nested with prominent plant-cell like cell membranes. Often mixed areas of classic ChRCC seen
b) Cytoplasm	Homogenously eosinophilic	Densely granular eosinophilic	Fine eosinophilic
c) Nucleus	Round to oval regular nuclei with focal perinuclear halo	Round and regular nuclei without perinuclear halo	Wrinkled nuclei with perinuclear halo
**IHC**	CD117 - Negative CK7 - Diffusely Positive	CD117 - Positive CK7 - Negative or focal positive.	CD117 - Positive CK7 - Negative or focal positive.
**Muller-Mowry Colloidal iron**	Negative or Apical bar, blob-like positive	Negative or only luminal pattern	Positive
**Electron microscopy**	Mitochondria excess	Mitochondria excess	Microvesicles along with mitochondria
**Karyotypic abnormality**	Del 1q, 19p and 19q	Del 1, 14 and y. 11q13 rearrangements and t(5;11) or no CNV	Either Del 1, 2, 6, 10, 13 and 17 or no CNV

**IHC: **Immunohistochemistry

Apart from describing clinicopathological and immunoprofiles, Trpkov et al. had aCGH performed in 9 of their cases (1). Frequent deletions in chromosomes 1q, 19p and 19q were observed. These karyotypic abnormalities are unique and different from those observed in eChRCC wherein either multiple losses of chromosome 1, 2, 6, 10, 13 and 17 (as in classic ChRCC) or no copy number variation (CNV) are seen (2).

HOCT with features mixed between RO and ChRCC is considered as a variant of ChRCC in the current 2016 WHO Classification (2). However, HOCT are usually diagnosed in the setting of multiple and bilateral tumors, typically in association with clinical scenarios of renal oncocytosis and Birt Hogg Dube syndrome (8,9). Definite diagnostic criteria for sporadic cases of HOCT are ambiguous and variable use of the terminology has been observed (4).

Clinically, all cases of LOT described by Trpkov et al. had indolent behavior with a favorable prognosis (1). However, precise biological behavior of these tumors could not be ascertained since the clinical follow up period was relatively limited. More studies with larger cohorts and longer follow up data are required to establish the indolent behavior of these tumors. The present case (Case 1) is disease free and is asymptomatic over a follow-up of 12 months, till date.

These two cases highlight that LOT can have different morphological patterns and a better understanding of the clinical, histomorphologic and immunoprofile of LOT is important to differentiate it from its mimickers with consequent development of management and surveillance guidelines. Also, as more cases are recognized and characterized, LOT would need to be formally incorporated as a provisional entity in the next WHO classification of renal tumors owing to its prognostic implications.

## Conflict of Interest

None of the authors have any conflict of interest

## Funding

None.

## Ethical Considerations

As per our Institutional Ethics Committee policy for case reports, consent is not required as long as the patient information is anonymized and the submission does not include images that may identify the person. In our case report, no patient identifying information or clinical images have been used. The authors state that the patient confidentiality has been maintained and no patient identifiers are used in this case report.
